# Physiological cyclic hydrostatic pressure induces osteogenic lineage commitment of human bone marrow stem cells: a systematic study

**DOI:** 10.1186/s13287-018-1025-8

**Published:** 2018-10-25

**Authors:** Elena Stavenschi, Michele A. Corrigan, Gillian P. Johnson, Mathieu Riffault, David A. Hoey

**Affiliations:** 1 Trinity Centre for Bioengineering, Trinity Biomedical Sciences Institute, Trinity College Dublin, Dublin 2, Ireland; 2 Department of Mechanical and Manufacturing Engineering, School of Engineering, Trinity College Dublin, Dublin 2, Ireland; 3 Department of Mechanical, Aeronautical and Biomedical Engineering, University of Limerick, Limerick, Ireland; 4 Advanced Materials and Bioengineering Research Centre, Trinity College Dublin and RCSI, Dublin 2, Ireland

**Keywords:** Mesenchymal stem cell, Bone, Mechanobiology, Osteogenic differentiation, Bioreactor

## Abstract

**Background:**

Physical loading is necessary to maintain bone tissue integrity. Loading-induced fluid shear is recognised as one of the most potent bone micromechanical cues and has been shown to direct stem cell osteogenesis. However, the effect of pressure transients, which drive fluid flow, on human bone marrow stem cell (hBMSC) osteogenesis is undetermined. Therefore, the objective of the study is to employ a systematic analysis of cyclic hydrostatic pressure (CHP) parameters predicted to occur in vivo on early hBMSC osteogenic responses and late-stage osteogenic lineage commitment.

**Methods:**

hBMSC were exposed to CHP of 10 kPa, 100 kPa and 300 kPa magnitudes at frequencies of 0.5 Hz, 1 Hz and 2 Hz for 1 h, 2 h and 4 h of stimulation, and the effect on early osteogenic gene expression of *COX2*, *RUNX2* and *OPN* was determined. Moreover, to decipher whether CHP can induce stem cell lineage commitment, hBMSCs were stimulated for 4 days for 2 h/day using 10 kPa, 100 kPa and 300 kPa pressures at 2 Hz frequency and cultured statically for an additional 1–2 weeks. Pressure-induced osteogenesis was quantified based on ATP release, collagen synthesis and mineral deposition.

**Results:**

CHP elicited a positive, but variable, early osteogenic response in hBMSCs in a magnitude- and frequency-dependent manner, that is gene specific. *COX2* expression elicited magnitude-dependent effects which were not present for *RUNX2* or *OPN* mRNA expression. However, the most robust pro-osteogenic response was found at the highest magnitude (300 kPa) and frequency regimes (2 Hz). Interestingly, long-term mechanical stimulation utilising 2 Hz frequency elicited a magnitude-dependent release of ATP; however, all magnitudes promoted similar levels of collagen synthesis and significant mineral deposition, demonstrating that lineage commitment is magnitude independent. This therefore demonstrates that physiological levels of pressures, as low as 10 kPa, within the bone can drive hBMSC osteogenic lineage commitment.

**Conclusion:**

Overall, these findings demonstrate an important role for cyclic hydrostatic pressure in hBMSCs and bone mechanobiology, which should be considered when studying pressure-driven fluid shear effects in hBMSCs mechanobiology. Moreover, these findings may have clinical implication in terms of bioreactor-based bone tissue engineering strategies.

**Electronic supplementary material:**

The online version of this article (10.1186/s13287-018-1025-8) contains supplementary material, which is available to authorized users.

## Background

Bone is exposed to constant cyclic loading which is necessary to maintain tissue integrity [1–4]. This effect is mediated in part, by bone marrow stem cells (BMSC), which undergo osteogenic lineage commitment in response to loading to replenish the population of bone-synthesising cells [5, 6]. Recently, it has been shown that bone marrow stem cells undergo osteogenesis in response to mechanical cues independent of osteocyte signalling, suggesting that the marrow micromechanical environment may directly influence BMSC osteogenesis [6–8]. Given the complex mechanical environment of bone, it is unclear how the translation of macro-scale mechanical cues to the marrow niche directly regulates stem cell differentiation. Fluid flow is recognised as the most potent biophysical stimulus contributing to bone anabolic responses [9–13]. However, the effect of pressure transients, which are necessary to drive the loading-induced fluid flow, on skeletal stem cell osteogenesis is poorly understood. Decoupling the effect of pressure transients from fluid shear on stem cell osteogenesis would identify the driving physical forces in loading induced bone formation, focusing efforts to utilise this information to enhance BMSC osteogenesis and bone repair.

The pressurisation of bone intraosseous fluid plays a critical role in bone mechanics as it provides hydraulic strengthening, as well as forcing the interstitial fluid and marrow, to flow through the lacunar-canalicular system (LCS) and within the medullary cavity, respectively [5, 14]. This, in turn, imparts fluid shear stress stimulation to the resident bone cells, in addition to enhancing mass transport and paracrine signalling [5, 15–18]. Under static conditions, the pressure generated within the marrow medullary cavity, known as intramedullary pressure (IMP), is approximately 4 kPa and related to the systemic blood pressure. However, fluctuations in IMP, which are pulsatile by nature, were found to be dependent on muscular contraction, the rate of loading and anatomic location, with magnitudes quantified up to 50 kPa [9, 19–21]. In addition, skeletal stem cells resident within Haversian channels and perivascular space may be exposed to pressures up to 300 kPa generated with loading within the LCS [5, 10, 22]. Pressure transients are paramount for the loading-induced bone anabolic response, yet it is not fully understood whether pressure plays a direct role in BMSC osteogenesis independent of the secondary fluid shear. This is of significant importance given that in vitro fluid flow bioreactors rely on pressure gradients to elicit dynamic flow, and these can vary depending on the inertial effects of fluid flow and geometry of the channels [11, 23–25].

One of the earliest studies quantifying the effect of pressure on ossifying long bones and calvaria rudiments demonstrated that cyclic hydrostatic pressure-enhanced mineral deposition, whereas continuous hydrostatic pressure had catabolic consequences [26]. Osteoblasts exposed to cyclic hydrostatic pressure (CHP, 10–40 kPa at 0.3–1 Hz) during short- and long-term mechanical stimulation exhibited temporal increases in bone-associated markers, as well as terminal osteoblastogenesis [24, 27–29]. Interestingly, osteoblasts treated with 68 kPa, 0.5 Hz CHP for an hour elicited an increase in the expression of the bone-associated marker, cyclo-oxygenase 2 (*Cox2*), whereas treatment of human bone marrow stem cells with a similar magnitude, but at 2 Hz frequency, did not mimic this response [24, 30]. In contrast, short-term administration of 10–36 kPa of hydrostatic or cyclic pressure (0.25 Hz) was sufficient to elicit an early osteogenic response in the expression of runt-related transcription factor 2 (*Runx2*), Osterix (*Osx*), distal-less homeobox 5 (*Dlx5*), Msh homeobox 2 (*Msx2*), bone morphogenetic protein 2 (*BMP2*) and alkaline phosphatase (ALP) in bone marrow-derived stem cells [31, 32]. Furthermore, 21-day treatment of 10 kPa hydrostatic pressure (HP) per day in osteogenic biochemical induction medium was sufficient to induce osteogenic lineage commitment of hBMSCs, whereas 90 kPa HP per day for 2 weeks after biochemical induction did not alter mineral deposition [31, 33]. Therefore, given the variable response to pressure in bone cells and limited analysis in human osteoprogenitors, a systematic study that investigates the effect of physiologically relevant pressure on osteogenic responses in human skeletal stem cells is required.

Therefore, the objective of this study is to conduct a systematic analysis of cyclic hydrostatic pressure magnitude, frequency and duration on early osteogenic responses and to determine whether these mechanical stimuli are sufficient to drive osteogenic lineage commitment of bone marrow-derived skeletal stem cells in the long term. Understanding how bone micromechanical cues modulate osteogenic hBMSCs potential, independent of fluid flow, may open new avenues for orthopaedic regenerative medicine strategies in addition to providing novel platforms to characterise loading induced skeletal stem cell osteogenesis.

## Methods

### Cell culture

All materials were purchased from Sigma-Aldrich unless otherwise stated. Human bone marrow-derived skeletal stem cells (hBMSCs) were isolated from bone marrow aspirates (Lonza) and characterised by trilineage differentiation (Additional file 1: Figure S1). hBMSCs were cultured on fibronectin (10 μg/ml, Corning) coated glass slides in low glucose DMEM supplemented with 10% foetal bovine serum (FBS, Biosera) and 1% penicillin-streptomycin (P/S) unless otherwise stated. For short-term mechanical stimulation, cells were cultured for 24 h under standard conditions followed by 48 h of serum starvation (0.5% FBS) supplemented with 10 nM dexamethasone, 0.025 mM L-ascorbic acid and 10 mM β-glycerol phosphate. These concentrations represent minimal levels for the support of osteogenesis, thereby allowing greater scope to investigate the effect of a biophysical versus a biochemical stimulus [11]. Regarding long-term cyclic hydrostatic pressure stimulation, the cells were cultured in similar conditions, except supplemented with 2% FBS and 2% P/S.

### Pressure bioreactor design

**Fig. 1:**
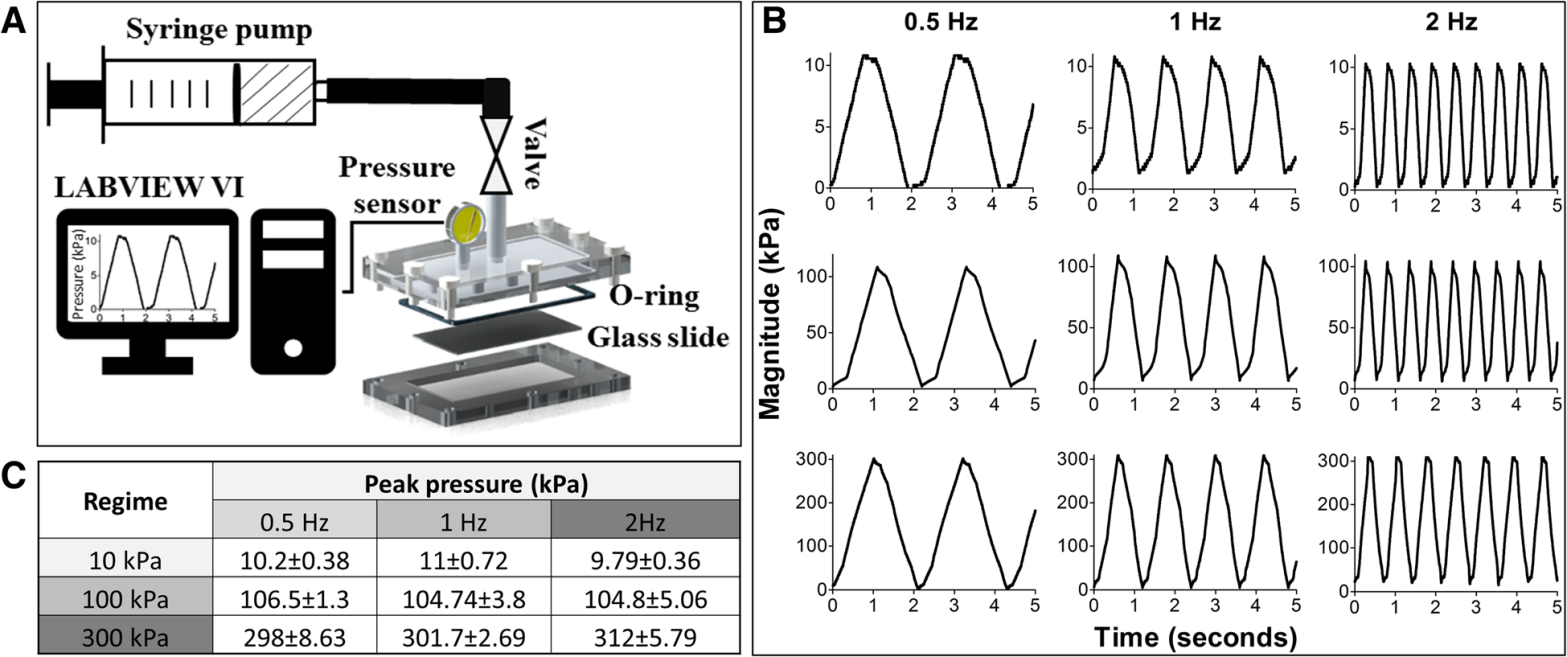
Pressure bioreactor design configuration (**a**) and validation of cyclic hydrostatic pressure regimes of 10 kPa, 100 kPa and 300 kPa at frequencies of 0.5 Hz, 1 Hz, 2 Hz. Representative traingular waveforms for each regime (**b**) and average peak pressures measured at room temperature represented as mean ± SD, *n* = 3 (**c**)

The design of the pressure bioreactor is based on the principle of fluid incompressibility whereby delivery of a finite bolus of fluid at various pressure rates in a closed system elicits a time-dependent pressure differential. The bioreactor system configuration is composed of a syringe pump (NE1660, New Era Pump Systems) which holds the syringe connected to the custom pressure bioreactor via tubing and a valve, a port on the pressure chamber for real-time pressure oscillations measurement (optional) and a pressure sensor (0–10 kPa: HSCMANV015PGAA5, 0–100 kPa: SSCDANV150PGAA5, Honeywell) interfaced with LABVIEW Virtual Instrument (Laboratory Virtual Instrument Engineering Workbench, National Instruments) to monitor and record pressure oscillations over time (Fig. 1a). The pressure bioreactor system was empirically validated for pressure magnitudes of 10 kPa, 100 kPa and 300 kPa at frequencies of 0.5 Hz, 1 Hz and 2 Hz. These pressure magnitudes have been predicted to occur within the bone marrow cavity and lacunar-canalicular system of bone [5, 10] whilst the employed frequencies are representative of human locomotion (Fig. 1b, c) [34, 35]. Given that fluid pressure is delivered to create a transient cyclic pressure differential, the shear stress was assumed to be negligible as volumes less than 2 ml were infused in a system of 50 ml of the medium. This was validated using blue dye to visualise the fluid streams during infusion/withdrawal phase where the dye reached only 1/3 of tubing and was not present within the chamber itself (data not presented). Hence, this allows for delineating the effect of pressure independent of fluid shear on the osteogenic commitment of hBMSCs.

### Cyclic hydrostatic pressure (CHP) mechanical stimulation

To systematically delineate the effect of cyclic hydrostatic pressure magnitude, frequency and duration on the early osteogenic response of hBMSCs, a series of CHP regimes were employed (Table 1). The range of pressure magnitudes selected represent the physiological pressures elicited within marrow just by muscle contraction independent of whole bone loading (~ 10 kPa) and pressure elicited within the lacunar canalicular system due to whole bone loading (300 kPa) [5, 21]. The frequencies of 0.5–2 Hz range are representative of human locomotion whereas the duration of mechanical stimulation is based on previous mechanobiology studies [11, 35, 36]. The CHP parameters were grouped such as to examine the independent effect of peak shear stress, frequency and duration of early osteogenesis. In addition, frequency and CHP duration were coupled such that comparisons of CHP regimes with a constant number of loading cycles can be made, i.e. 0.5 Hz, 4 h against 1 Hz, 2 h and 2 Hz, 1 h. This approach is based on a previous systematic investigation of oscillatory fluid flow on mesenchymal stem cells [11]. The static condition for both short- and long-term stimulation consisted of culture slides assembled within chambers and were left open to atmospheric pressure (control). To determine whether cyclic hydrostatic pressure can induce osteogenic lineage commitment, hBMSCs were subjected to three separate CHP regimes over a long-term duration, based on our results from the short-term systematic analysis. The three chosen CHP regimes of 10 kPa, 2 Hz, 100 kPa, 2 Hz and 300 kPa, 2 Hz were applied intermittently on days 3, 5, 7 and 9 for 2 h/day and subsequently cultured statically for an additional 7 and 14 days (experimental plan illustrated in Additional file 1: Figure S2).

**Table 1:** Experimental conditions for short-term fluid pressure stimulation

Shear stress	Frequency			Infused volume/cycle (ml)
	0.5 Hz (h)	1 Hz (h)	2 Hz (h)	
10 kPa	2,4	2	1,2	≤ 0.03
100 kPa	2,4	2	1,2	≤ 0.375
300 kPa	2,4	2	1,2	≤ 1.5

### Quantitative real-time PCR

Cells were lysed using TRI Reagent® (Sigma Aldrich), and mRNA was isolated according to the manufacturer’s protocol. One microgram of RNA was reverse transcribed into cDNA using High Capacity cDNA kit (Life Technologies). Quantitative polymerase chain reaction (qPCR) was performed using SYBR Select Mastermix (ThermoFisher 4472903). The expression of 18S ribosomal RNA (*18S*), glyceraldehyde 3-phosphate dehydrogenase (*GAPDH*), cyclooxygenase 2 (*COX2*), runt-related transcription factor 2 (*RUNX2*) and OSTEOPONTIN (*OPN*) were quantified using primers detailed in Table 2 (Sigma Aldrich). The amplification was performed with an ABI7500 Fast Real-Time PCR machine. Each sample was normalised to reference genes *18S* and *GAPDH* and to static control using relative quantification method.

**Table 2:** Primers and experimental conditions used for qPCR analysis

Gene	Tm (°C)	Primer concentration (nM)	Sequence	PCR product size (bp)
*18S*	60	300	5′- ATCGGGGATTGCAATTATTC -3′	130
			3′- CTCACTAAACCATCCAATCG -5′	
*GAPDH*	60	300	5′- ACAGTTGCCATGTAGACC -3′	95
			3′- TTTTTGGTTGAGCACAGG -5′	
*COX2*	60	400	5′- GGAGAAAAGGAAATGTCTGC -3′	186
			3′- GTAGGCAGGAGAACATATAAC -5′	
*RUNX2*	60	400	5′- GCAGTATTTACAACAGAGGG -3′	112
			3′- TCCCAAAAGAAGTTTTGCTG -5′	
*OPN*	60	400	5′- GACCAAGGAAAACTCACTAC -3′	84
			3′- CTGTTTAACTGGTATGGCAC -5′	

### Adenosine triphosphate assay

After mechanical stimulation for each time point (1, 2 and 4 h for days 3, 5, 7 and 9), the cells were incubated in 1 ml of medium after which the medium was collected and snap frozen in liquid nitrogen following storage at − 80 °C. Adenosine triphosphate (ATP) metabolites within the media were measured using Molecular Probes® ATP Determination Kit (A22066, Invitrogen) according to the manufacturer’s protocol. Luminescence was measured using Luminoskan™ Ascent Microplate Luminometer (MTX LAB SYSTEMS).

### Osteogenic assays

Cells were fixed in formalin for 10 min. For collagen staining, cells were incubated in 0.1% Picro-Sirius Red solution for 1 h at room temperature. After washing twice in 0.5% acetic acid and water, samples were mounted with DPX mounting medium. Calcium staining was performed using 2% Alizarin Red solution. The bound dye for both calcium and collagen was observed under light microscopy. The bound Alizarin Red was used to quantify the calcium content by extraction using 10% *v*/*v* acetic acid and measuring the absorbance at 405 nm.

### Statistical analysis

All data is presented as mean ± SEM. For qRT-PCR analysis, each condition was compared to matched static control using a two-tailed student’s *t*-test with Welch’s correction. CHP to static control (C) is denoted as *p < 0.05, **p < 0.01 and ***p < 0.005. One-way ANOVA with the Tukey post hoc test was used to compare the effect of magnitude, frequency and duration, and significant differences are indicated as ^#^*p* < 0.05, ^##^*p* < 0.01 and ^###^ *p* < 0.005 except for the 2-h group, where a two-way ANOVA analysis was employed with the Bonferroni post hoc test. For calcium quantification, a one-way ANOVA with Dunnett’s post-test was performed.

## Results

### Cyclic hydrostatic pressure bioreactor design and validation

The design of the pressure bioreactor system was modelled on our previously developed fluid shear bioreactor due to its ease of use and the compatibility of glass slides for cell culture and syringe pumps for mechanical stimulation (Fig. 1a). The custom pressure bioreactor was designed to allow for pressure stimulation of cells seeded on 2D substrates (i.e. glass or polydimethylsiloxane), in addition to cells seeded on three-dimensional scaffolds. By harnessing the power of fluid incompressibility, cyclic pressure transients can be achieved by applying a cyclic oscillatory fluid flow of finite volumes of fluid, in a closed system filled with culture medium. Using an external port which allows for real-time measurement of pressures, the pressure bioreactor was validated to generate pressure transients of 10 kPa, 100 kPa and 300 kPa at frequencies of 0.5 Hz, 1 Hz and 2 Hz with a triangular waveform (Fig. 1b). The average peak pressures achieved using this system are within 15% of nominal pressures.

### Effect of CHP magnitude on early osteogenic gene expression in hBMSCs

**Fig. 2:**
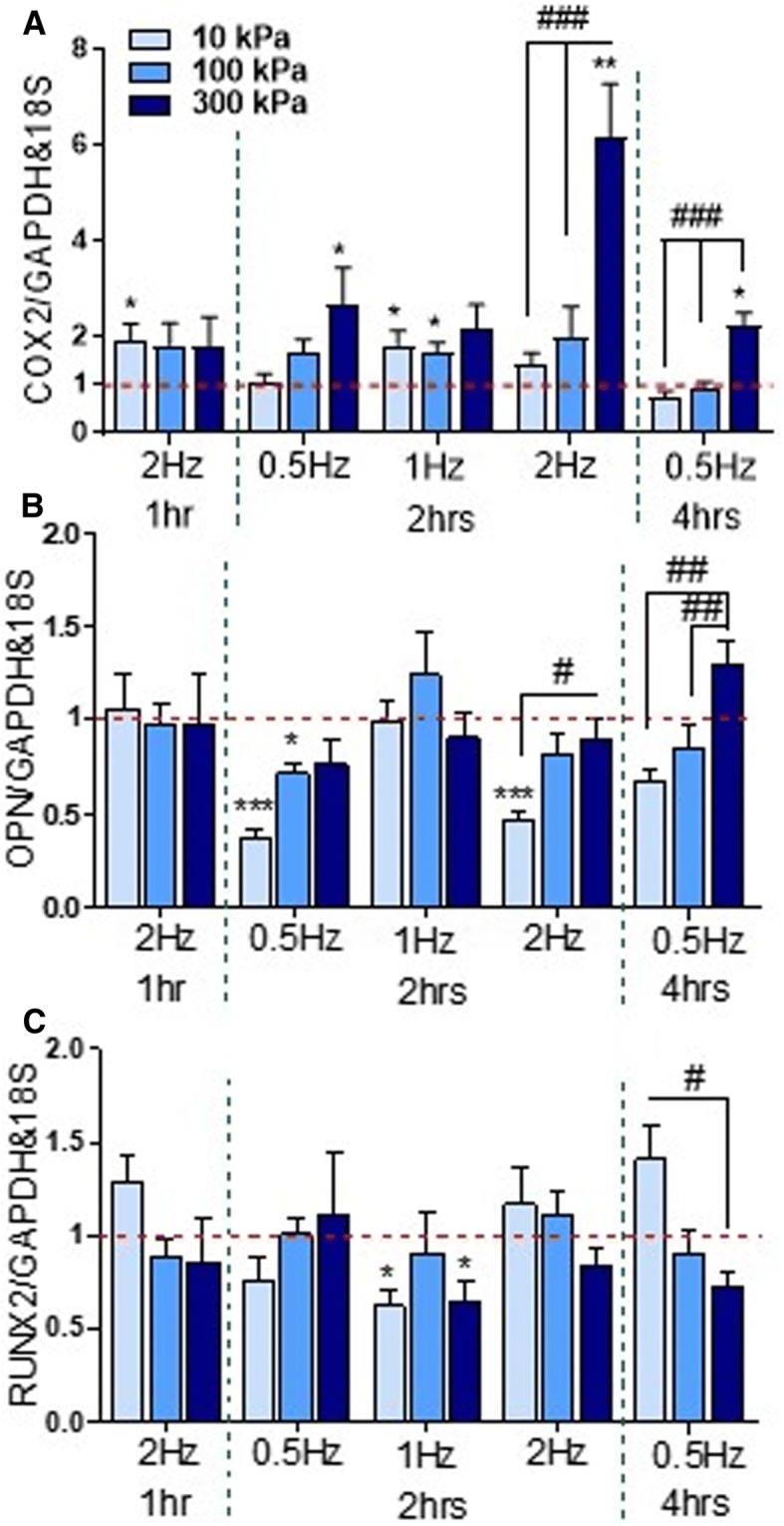
**a**–**c** Effect of cyclic hydrostatic pressure magnitudes of 10 kPa, 100 kPa and 300 kPa on early osteogenic gene expression in human skeletal stem cells at frequencies of 0.5 Hz, 1 Hz and 2 Hz. *N* = 2, *n* = 3–4. *p < 0.05, ***p < 0.001 compared to static control; ^#^*p* < 0.05, ^##^*p* < 0.01, ^###^*p* < 0.001 for magnitude effect

Stimulation of hBMSCs with cyclic hydrostatic pressure displays a variable osteogenic response based on mRNA expression of the osteogenic markers *COX2*, *RUNX2* and *OPN* compared to static conditions. *COX2* mRNA expression is upregulated in response to CHP in a magnitude dependent manner (Fig. 2a). This is particularly evident in the 0.5 Hz, 2 h, 0.5 Hz, 4 h and 2 Hz, 2 h groups, significant only for the two latter groups (*p* < 0.001). In addition, the highest expression within these groups is achieved at 300 kPa magnitude and is significantly different to 10 kPa and 100 kPa (*p* < 0.05). Interestingly, for the 2 Hz, 1 h and 1 Hz, 2 h groups, a consistent approximately twofold change is maintained, displaying little effect of magnitude. This may be attributed to a synergistic time effect as significant magnitude effects are present at a longer duration of stimulation. Overall, CHP did not cause any changes in RUNX2 expression, except an inhibitory magnitude effect was present at the 0.5 Hz, 4 h time point (*p* < 0.05) and a significantly decreased expression at 10 kPa and 300 kPa at 1hz, 2 h (*p* < 0.05) (Fig. 2b). *OPN* mRNA expression displayed an inhibitory effect at lower magnitudes of CHP, with a positive magnitude effect for the 2 Hz, 2 h and 0.5 Hz, 4 h groups (*p* < 0.05) (Fig. 2c).

### Effect of CHP frequency on early osteogenic gene expression in hBMSCs

**Fig. 3:**
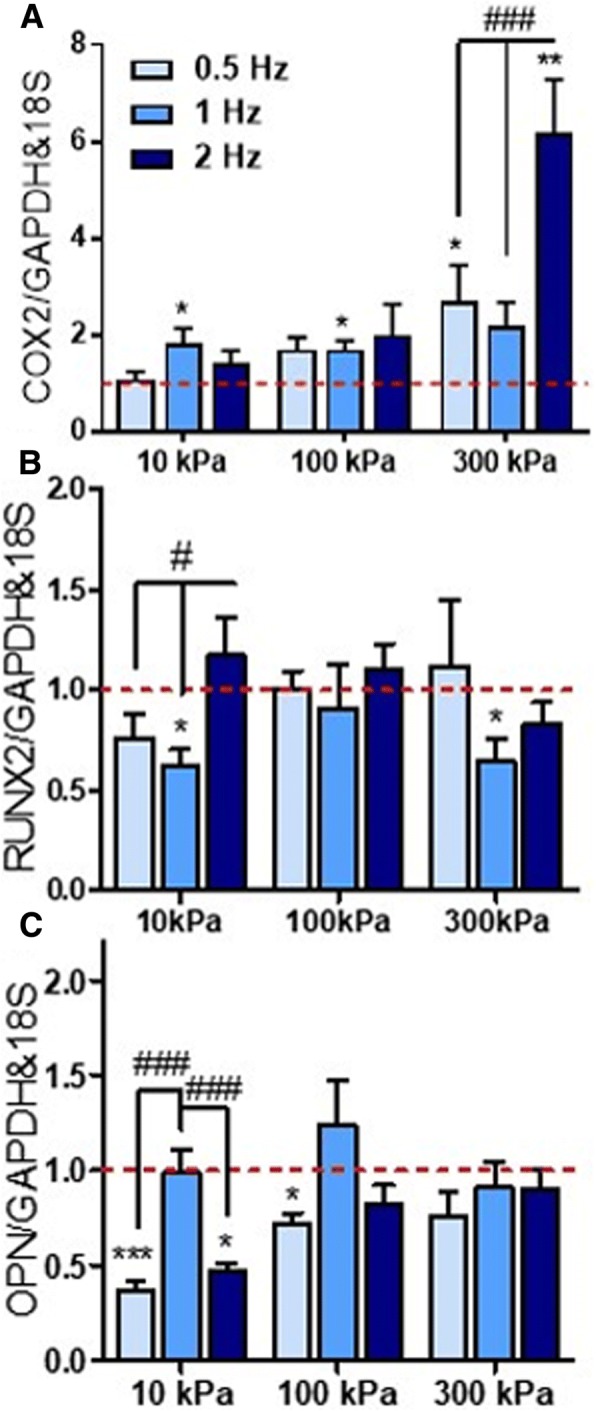
**a**–**c** Effect of CHP frequency (0.5 Hz, 1 Hz and 2 Hz) on early osteogenic gene expression for 10 kPa, 100 kPa and 300 kPa pressure magnitudes (2 h duration). *N* = 2, *n* = 3–4. *p < 0.05, ***p < 0.001 compared to static control; ^#^*p* < 0.05, ^##^*p* < 0.01, ^###^*p* < 0.001 for magnitude effect

To identify whether CHP frequency affects early osteogenic mRNA expression, the 2 h pressure group was analysed for 0.5 Hz, 1 Hz and 2 Hz frequencies. Pressure-induced *COX2* mRNA expression displays a similar level of upregulation at all magnitudes irrespective of frequencies, except at 300 kPa, where a frequency effect is present (*p* < 0.001) (Fig. 3a). Interestingly, for *RUNX2* and *OPN* mRNA expression, a frequency effect is present only at 10 kPa magnitude (*p* < 0.05). Specifically, *OPN* mRNA expression at 10 kPa was least inhibitory at 1 Hz compared to 0.5 Hz and 2 Hz (*p* < 0.001). This effect is also present at 100 kPa, although not significant for frequency effects. Hence, the frequency of pressure stimulation plays a role at higher magnitudes for *COX2* expression and at lower magnitudes for *RUNX2* and *OPN* mRNA expression.

### Effect of CHP duration on early osteogenic gene expression in hBMSCs

**Fig. 4:**
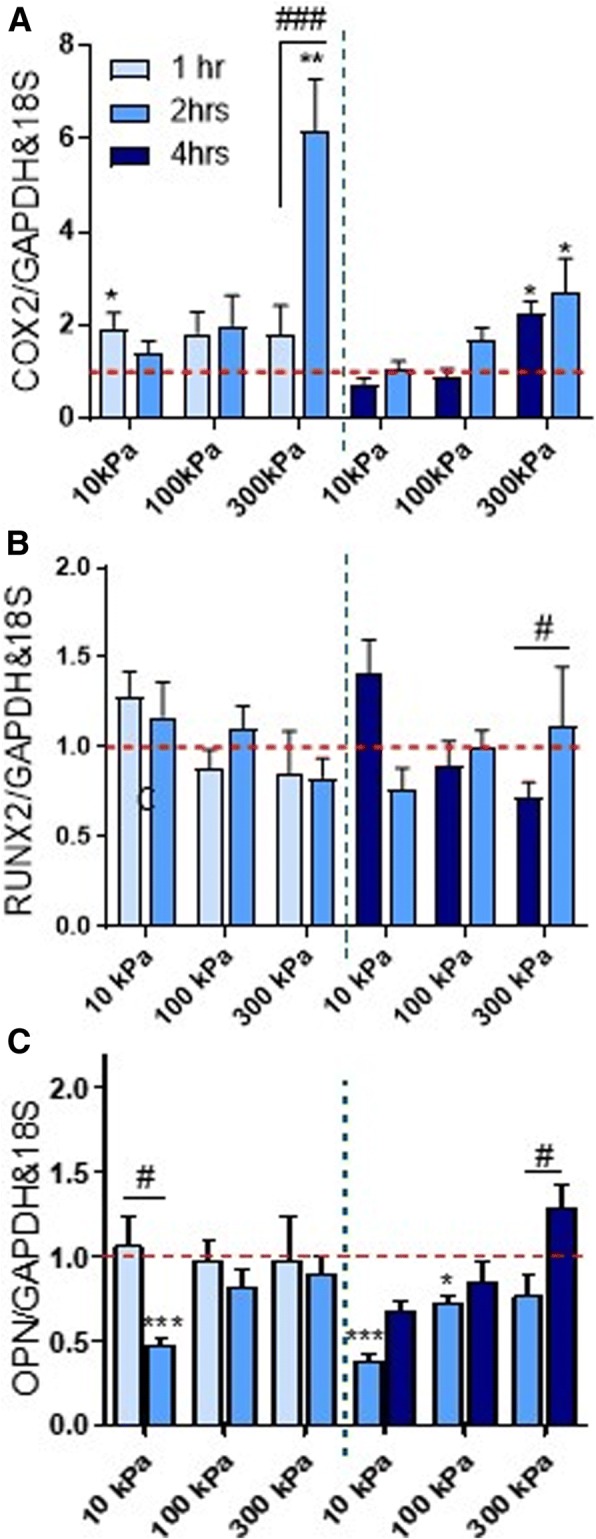
**a**–**c** Effect of duration of CHP stimulation on early osteogenic gene expression at frequencies of 0.5 Hz (2 and 4 h) and 2 Hz (1 and 2 h). *N* = 2, *n* = 3–4. *p < 0.05, ***p < 0.001 compared to static control; ^#^*p* < 0.05, ^##^*p* < 0.01, ^###^*p* < 0.001 for magnitude effect

To identify the effect of duration on early osteogenic expression, 1 h and 2 h, as well as 2 h and 4 h of stimulation, were compared while other parameters were held constant. Overall, *COX2* mRNA expression displayed similar levels of upregulation over time, except at 300 kPa, 2 Hz frequency where a higher response was observed when stimulated for longer durations (*p* < 0.001) (Fig. 4a). In contrast, *RUNX2* mRNA expression displayed no changes over time for the compared groups, except at 300 kPa where a significant decrease at 4 h versus 2 h was noted (*p* < 0.05). When comparing the 1 h, 2 Hz with 2 h, 1 Hz and 4 h, 0.5 Hz groups, all of which experience the same cyclic hydrostatic pressure magnitude and number of oscillating cycles (7200), there is a clear abrogated response with the longer duration for 10 kPa and 300 kPa suggesting that *RUNX2* expression may have a time dependency effect (Fig. 4b). No changes over time were observed for *OPN* mRNA expression except an inhibitory effect at 10 kPa at 2 h versus 1 h (*p* < 0.05) and a significant upregulation at 4 h versus 2 h at 300 kPa (*p* < 0.05) (Fig. 4c). Therefore, duration of mechanical stimulation plays a role in the level of upregulation of osteogenic markers although this effect is gene and magnitude dependent.

### Effect of long-term intermittent CHP on the osteogenic lineage commitment of hBMSCs

**Fig. 5:**
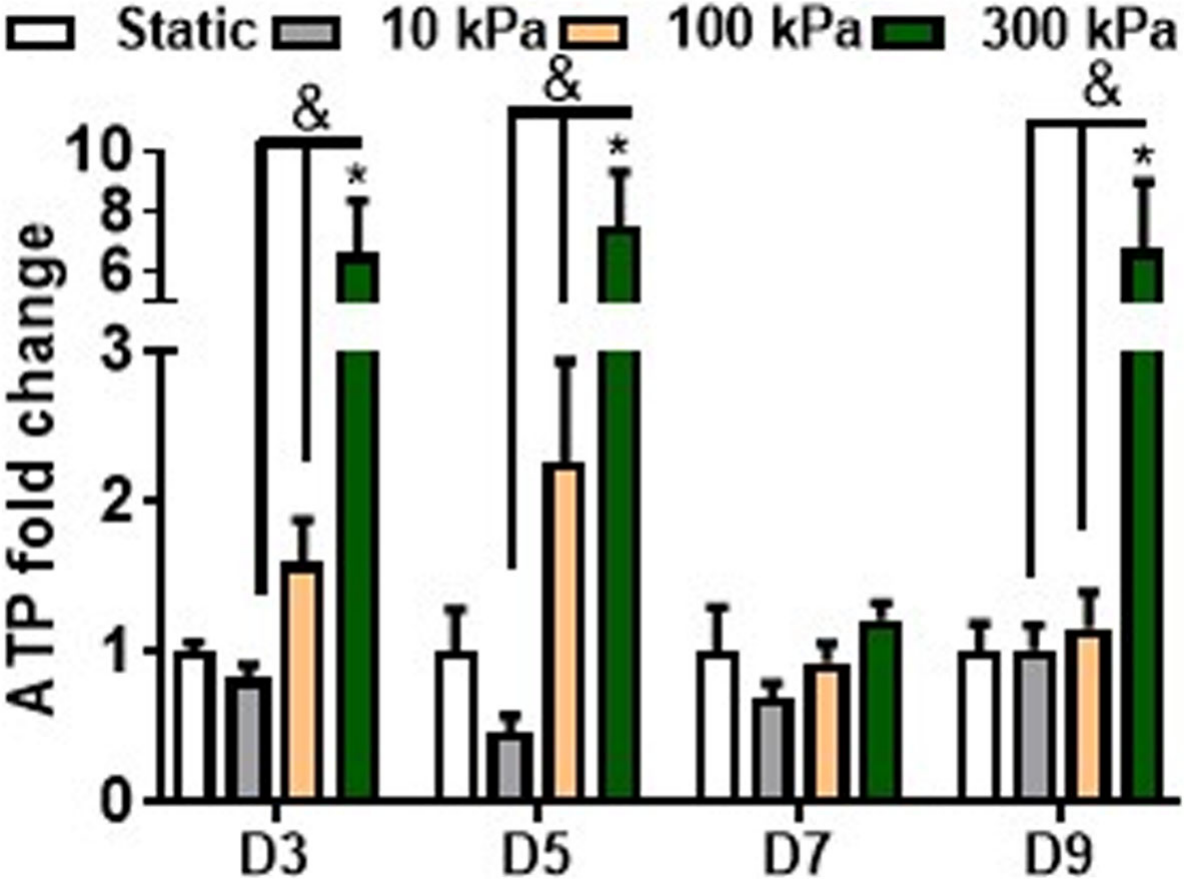
ATP release over time during long-term intermittent pressure stimulation. The effects of intermittent pressure on ATP release at days 3, 5, 7 and 9 after 1 h of mechanical stimulation. *n* = 4–6. *p < 0.05 compared to either S; ^&^*p* < 0.05 for effect of pressure magnitude effect

Based on the systematic analysis performed above, where the most robust osteogenic gene expression was observed at 2 Hz frequency for all pressure magnitudes, these three pressure regimes were brought forward to verify whether CHP can induce the osteogenic lineage commitment of hBMSCs. Therefore, the pressure regimes of 10 kPa, 100 kPa and 300 kPa at 2 Hz frequency were applied once per day for a total of 4 days followed by 1 and 2 weeks of static culture. Furthermore, to determine whether the hBMSCs were responsive throughout the long-term CHP stimulation, ATP release after 1 h of stimulation was determined on each day of pressure stimulation (Fig. 5). ATP release within the media of the stimulated hBMSCs when compared to static controls, displayed a pressure magnitude effect (*p* < 0.05) for all days except day 7. Ten-kilopascal pressure did not induce ATP release at any time point. There was a trend towards significance at 100 kPa at day 3 and day 5 (*p* < 0.07 at day 3, *p* < 0.14 at day 5), while there was a consistent increase in ATP release following 300 kPa pressure (*p* < 0.05) at days 3, 5 and 9. The continuous ATP release over-time confirms that hBMSCs maintained their mechanoresponsiveness over extended periods of pressure stimulation and confirms the magnitude effect of pressure stimulation demonstrated above.

**Fig. 6:**
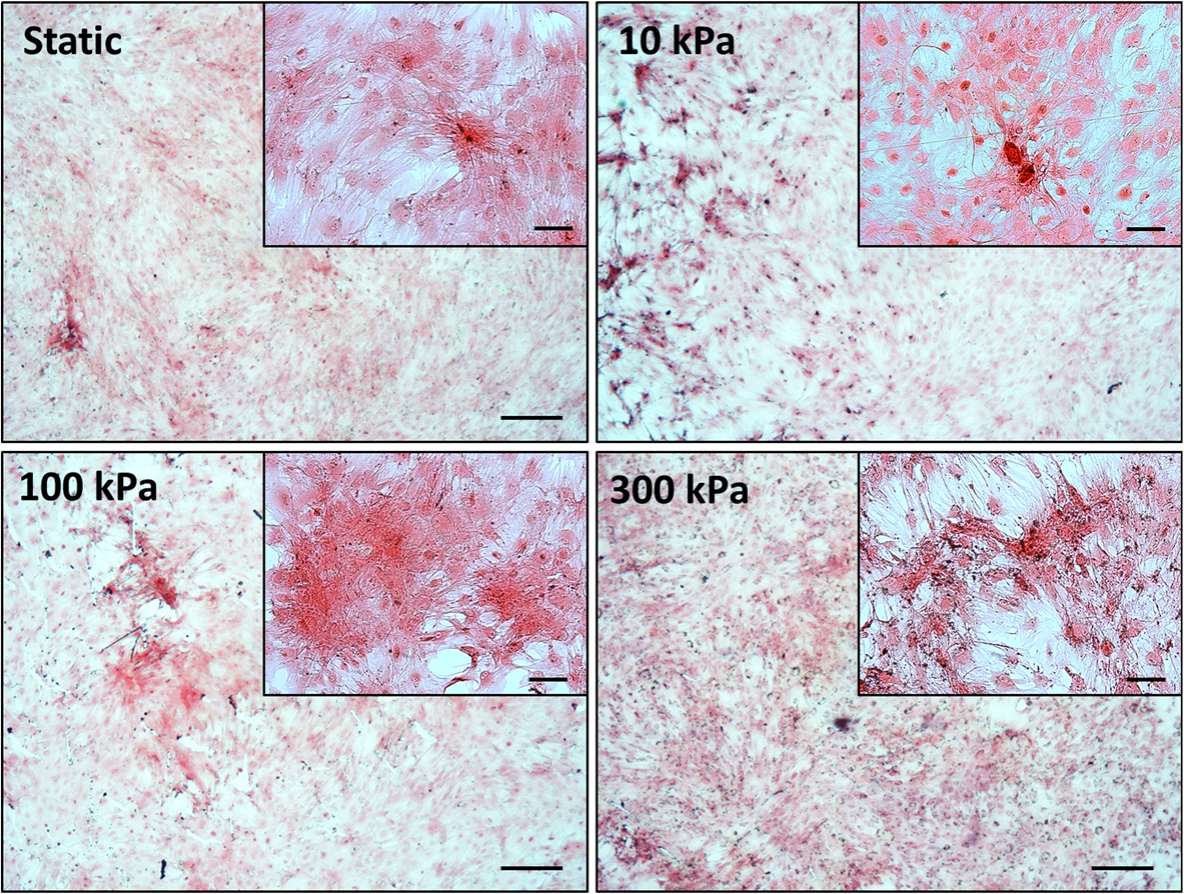
PicroSirius Red staining for collagen after long term pressure stimulation (4 days loading and 17 days static). Static—static condition in minimum osteogenic conditions, pressure mechanical stimulation in minimum osteogenic conditions at 10 kPa, 100 kPa and 300 kPa at 2 Hz frequency. Scale bar = 250 μm

**Fig. 7:**
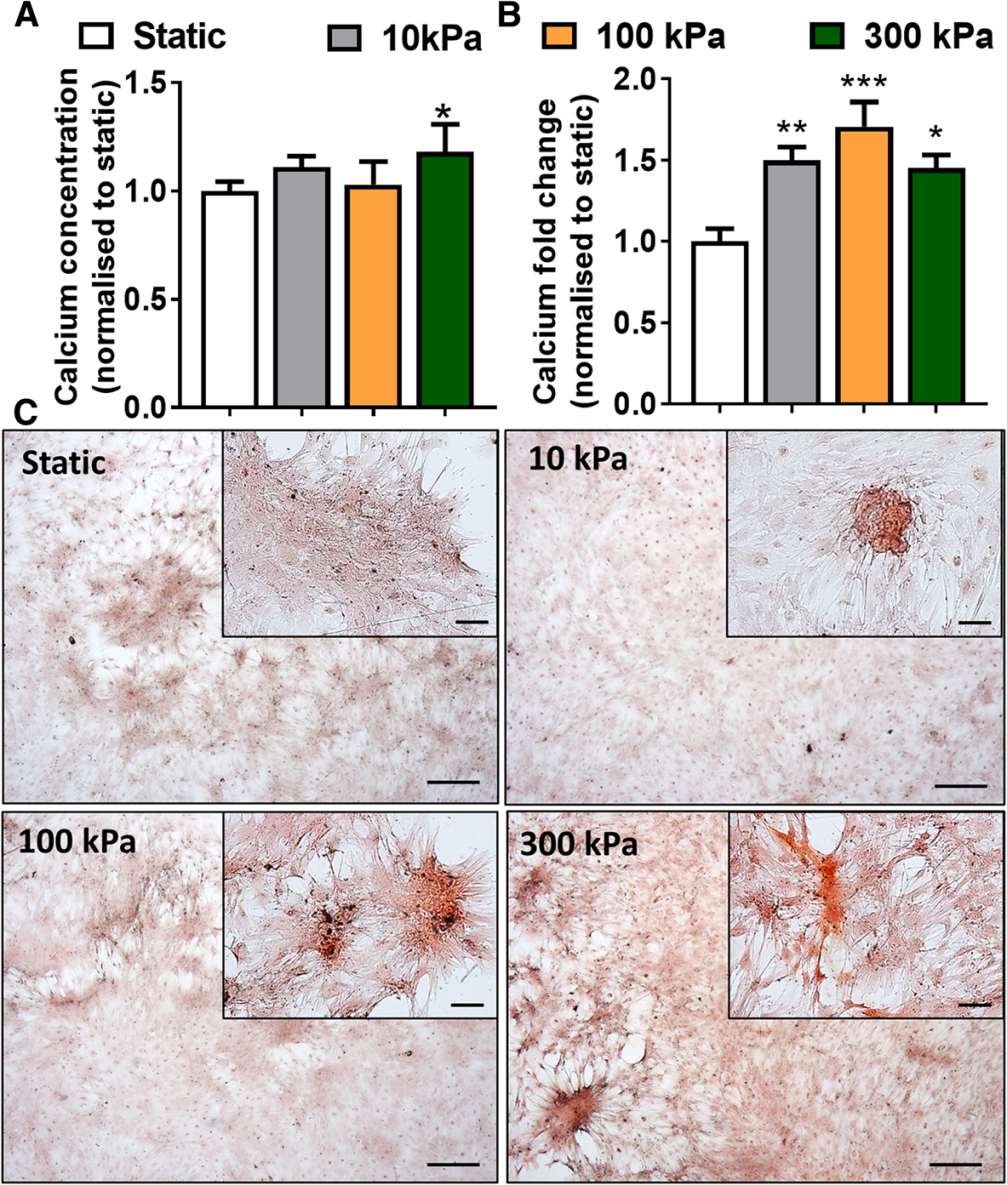
Cyclic hydrostatic pressure stimulation induces mineralisation of hBMSCs. A) Calcium concentration after 4 days of CHP + 10 days static culture (**a**) and 4 days of CHP + 17 days static culture (**b**). Alizarin S staining for mineralisation after long term pressure stimulation show presence of mineral nodules with CHP (4 days CHP + 17 days static culture) (**c**). Static—static condition in minimum osteogenic conditions, pressure mechanical stimulation in minimum osteogenic conditions at 10 kPa, 100 kPa and 300 kPa at 2 Hz frequency. Scale bar = 250 μm. *p < 0.05, **p < 0.01, ***p < 0.001

After relatively short-term CHP treatment (4 days), followed by a 2-week static culture, collagen synthesis is observed in all groups. However, the pressure stimulated groups displayed regions of increased collagen deposition when compared to static conditions (Fig. 6). No distinct qualitative differences in collagen synthesis are noted between the CHP groups. Regarding mineralisation1-week post CHP stimulation, there is a slight but significant increase in mineral deposition for the high magnitude 300 kPa, 2 Hz group (*p* < 0.01) (Fig. 7a). However, at 2 weeks post pressure stimulation, quantification of Alizarin Red S staining shows a significant increase in calcium deposition with CHP in all groups (*p* < 0.05), with no difference between each magnitude of pressure. The presence of mineralised nodules is detected for all the CHP regimes as illustrated in Fig. 7c. Therefore, physiologically relevant cyclic hydrostatic pressures predicted to occur within the bone and marrow cavity is sufficient to directly drive osteogenic lineage commitment of human stem cells independent of pressure magnitude in the long term, despite demonstrating early magnitude dependent effects.

## Discussion

We have previously demonstrated that physiological levels of fluid flow that are predicted to occur within bone marrow niche, drive stem cell osteogenic lineage commitment in a shear stress and magnitude dependent manner [11]. However, the effect of pressurisation of the intraosseous fluid, which drives this fluid flow, and its effects independent of fluid shear on stem cell osteogenesis is poorly understood. For the first time, we report a systematic in vitro analysis on the effect of physiological CHP magnitude, frequency and duration on human bone marrow stem cell osteogenic responses. Through the utilisation of custom-built pressure bioreactors, we demonstrated that CHP elicits a variable, yet, positive osteogenic response in hBMSCs in a magnitude, frequency, and duration-dependent manner, that is gene specific. Furthermore, long-term mechanical stimulation utilising 10–300 kPa pressure magnitudes at 2 Hz frequency promoted collagen synthesis and significant mineral deposition compared to static control, proving that physiological levels of pressure elicited within the marrow and bone with loading is sufficient to directly drive osteogenic lineage commitment. Moreover, mineral deposition due to CHP stimulation was found to be independent of pressure magnitude, despite early osteogenic magnitude dependent effects. These findings highlight that bone marrow physiological CHP can directly drive stem cell osteogenesis, independent of fluid flow, which should be considered when studying pressure driven fluid shear effects in hBMSC mechanobiology. Moreover, these findings may open new avenues for orthopaedic regenerative medicine strategies, in addition to providing novel platforms to characterise loading induced skeletal stem cell osteogenesis.

Human BMSCs were found to be mechanosensitive to pressure stimuli and elicited magnitude, frequency and duration dependent osteogenic responses to physiological bone CHP. *COX2* and *OPN* mRNA expression were found to be magnitude dependent at 2 Hz, 2 h and 0.5 Hz, 4 h whereas *RUNX2* expression did not demonstrate this dose-dependent effect, possibly highlighting activation of alternative pathways. Moreover, when normalising for the number of oscillations comparing 1 Hz, 2 h versus 2 Hz, 1 h (120 oscillations), *COX2* and *OPN* mRNA expression is similar and independent of pressure magnitude, whereas, when doubling the amount of oscillations at 2 Hz, 2 h and 0.5 Hz, 4 h (240 oscillations) the magnitude effects become significant. This may indicate that for magnitude dependent effects a cumulative threshold of stimulation must be reached for differential expression of osteogenic genes to be elicited. *COX2* expression was shown to be sensitive to the magnitude of fluid flow stimulation in both osteocytes and stem cells and its expression has been highlighted as a precursor for osteoblastogenesis [11, 36, 37]. Pressure-induced *COX2* expression was found to be elicited in osteocytes (68 kPa, 0.5 Hz) and osteoblasts (68 kPa, 0.5 Hz) but not hBMSCs (50 kPa, 2 Hz), which may indicate that the pressure response was either frequency or lineage dependent [24, 30, 38]. Given that CHP induced a magnitude dependent response in our experimental setup, this could be attributed to the prior biochemical priming of hBMSC for 2 days before mechanical stimulation. However, we have also observed pressure induced *Cox2* upregulation in a mesenchymal stem cell line (MSC), C3H10T1/2 using the same system, highlighting the possibility in the difference of the bioreactor set up and mode of pressurisation in other studies (data not shown).

Interestingly, the overall expression of *OPN* seemed unaltered or inhibited when compared to static culture, with several exceptions of positive magnitude effect at 0.5 Hz and 2 Hz frequencies. Changes in *OPN* mRNA levels have been reported to depend on the stage of osteogenic differentiation, as osteoprogenitor cells were found to exhibit lower basal levels and no changes in *Opn* mRNA expression in response to 13 kPa, 0.3 Hz CHP when compared to late-stage osteoblasts [27]. Similarly, the observation that the master transcription factor *RUNX2* remained unaltered or downregulated in some CHP regimes, may indicate that either CHP inhibits osteogenesis or alternatively results in changes in the translation of the protein, as has been previously shown in osteoblasts using a cyclic stretch model [39]. Interestingly, CHP was previously shown to stimulate collagen synthesis and bone mineral deposition in chick femurs, although no changes in *Runx2* expression were observed [40]. Similar to our observations, pressure induced osteogenic lineage commitment of hBMSCs, in spite of unaltered *RUNX2* expression, alluding to the latter case of changes in protein translational activity.

Pressure induced an early osteogenic response in hBMSC showing frequency dependent effects which were magnitude-gene coupled. Specifically, *COX2* mRNA expression displayed a frequency dependent effect at 300 kPa magnitude, whereas *RUNX2* and *OPN* at 10 kPa CHP. Interestingly, fluid flow induced *Cox2* mRNA expression in both MSCs and osteocytes was found to have weak frequency effects, except at high magnitudes, demonstrating this mechanically driven *COX2* response is consistent across all forms of mechanical stimulation. Increase in bone formation due to CHP stimulation in chick femurs was found to be proportional to frequency but independent of pressure magnitude applied [40]. Similarly, 2 Hz was found to be the optimum frequency for loading-induced mineralisation in an osteoblast model of dynamic compression [41]. This effect may be attributed to a universal magnitude-frequency response, possibly related to human kinematics and loading-induced bone formation [11, 36, 42, 43].

During long-term stimulation, using the most pro-osteogenic magnitude dependent 2 Hz frequency regime, we observed that hBMSC were mechanoresponsive to CHP loading overtime by secreting ATP metabolites into the medium. Moreover, this ATP release was magnitude depedent over time at each loading event. To date, only osteoblasts have been shown to elicit an ATP response to CHP, whereas, stem cells were found to secrete ATP in a fluid flow magnitude dependent manner [44–46]. Purinergic signalling plays a crucial role in bone anabolism, therefore ATP synthesis in response to mechanical stimulation may highlight initiation of mechanotransduction events, irrespective of type of stimulus [47–49]. Although hBMSCs display a pressure magnitude dependent sensitivity for ATP release, this did not correlate with the ability to synthesise bone mineral indicating that other mechanisms may be at play.

Cyclic hydrostatic pressure induces osteogenic lineage commitment of skeletal stem cells independent of magnitude of stimulation. This supports various reports, where pressure induced mineralisation in osteoblasts and stem cells, was shown to be modulated in vitro at low (< 40 kPa) and high (200 kPa) magnitudes, either statically or dynamically [27–29, 31, 50, 51]. Ex vivo intramedullary pressurisation of ulnae, independent of matrix deformation, showed a positive correlation between transcortical pressure gradients and enhanced bone formation. This effect was associated to fluid flow, as transcortical pressure gradients are related to fluid flow velocity [10]. Similarly, magnitude of shear stress, at the same frequency (2 Hz) of CHP stimulation, was shown to elicit a positive effect on mineralisation in stem cells [11]. Biomechanically, bone deformation causes pressurisation of intraosseous fluid which forces it to flow within the bone. Given the incompressibility of the water based intraosseous fluid, small pressure gradients can elicit large inertial fluid effects due to the architecture of bone tissue. From this perspective, and the biological effects reported above, it can be concluded that pressure gradients play a role in hBMSCs osteogenesis, but this effect may be secondary to fluid flow. However, since fluid flow bioreactors rely on pressure driven flow that at often times reach pressure differentials higher than 10 kPa, these pressure effects should be accounted for in mechanobiology studies. A limitation of this study is that only the 2 Hz frequency CHP regime was used for long term pressure induced osteogenesis, hence these effects may be specific to this frequency alone.

## Conclusion

In conclusion, we demonstrate that hBMSCs are mechanosensitive to pressure stimuli with a magnitude-dependent *COX2* mRNA expression and ATP-associated purinergic signalling. Although *RUNX2* and *OPN* mRNA expression remained unaltered with short-term CHP stimulation, application of physiologically associated cyclic hydrostatic pressures, over a long term, was found to induce osteogenic lineage commitment of hBMSCs, which was independent of pressure magnitude and frequency. This highlights that physiologically low marrow pressures can also affect the hBMSCs osteogenic potential, similar to fluid flow; however, its effects may be secondary. The systematic approach taken enabled the identification of early pressure differential effects which can be used to delineate the mechanisms of pressure induced osteogenesis. This can also serve as a platform to discover novel targets for bone therapies [31]. Moreover, these findings may support the use of mechanotherapies in clinical applications for patients displaying osteopenia/bone fragility, in addition to optimisation of bioreactor design for bone regenerative strategies.

## Additional file

Additional file 1: **Figure S1.** Validation of trilineage potential of hBMSC for adipogenesis (Oil red O, A), chondrogenesis (Alician Blue, B) and osteogenesis (Alizarin Red S, C) after 21 days in culture. Zoomed in images point to triglyceride accumulation in adipogenic conditions. Scale bar = 200 μm. Figure S2 Schematic of long term pressure mechanical stimulation regime. (DOCX 600 kb)

## Abbreviations

18s: 18s ribosomal RNA
*ALP*: Alkaline phosphotase
ATP: Adenosine triphosphate
*BMP2*: Bone morphogenetic protein 2
BMSC: Bone marrow-derived stem cells
cDNA: Circular deoxyribonucleic acid
CHP: Cyclic hydrostatic pressure
Cox2: Cyclooxygenase 2
Dlx5: Distal-less homeobox 5
DMEM: Dulbecco’s modified Eagle medium
FBS: Fetal bovine serum
GAPDH: Glyceraldehyde 3-phosphate dehydrogenase
hBMSC: Human bone marrow-derived stem cells
HP: Hydrostatic pressure
IMP: Intramedullary pressure
LABVIEW: Laboratory Virtual Instrument Engineering Workbench
LCS: Lacunar canalicular system
mRNA: Messenger ribonucleic acid
MSC: Mesenchymal stem cells
Msx2: Msh homeobox 2
nM: Nanomolar
Opn: Osteopontin
Osx: Osterix
P/S: Penicillin streptomycin
qPCR: Quantitative polymerase chain reaction
RNA/rna: Ribonucleic acid
Runx2: Runt-related transcription factor 2
