# Supplementary material for: Physiological cyclic hydrostatic pressure induces osteogenic lineage commitment of human bone marrow stem cells: a systematic study
Source: Stem Cell Res Ther. 2018 Oct 25;9:276. doi: 10.1186/s13287-018-1025-8 (PMC6203194; doi:10.1186/s13287-018-1025-8)
Supplement: Supplementary file 1 — Figure S1. Validation of trilineage potential of hBMSC for adipogenesis (Oil red O, A), chondrogenesis (Alician Blue, B) and osteogenesis (Alizarin Red S, C) after 21 days in culture. Zoomed in images point to triglyceride accumulation in adipogenic conditions. Scale bar = 200 μm. Figure S2 Schematic of long term pressure mechanical stimulation regime. (DOCX 600 kb) [file 13287_2018_1025_MOESM1_ESM.docx]

# Physiological cyclic hydrostatic pressure induces osteogenic lineage commitment of human bone marrow stem cells: a systematic study

Elena Stavenschi^1,2^, Michele A. Corrigan^1,2^, Gillian P. Johnson^1,2,3^, Mathieu Riffault^1,2,4^, David A. Hoey^1,2,3,4^

^1^Trinity Centre for Bioengineering, Trinity Biomedical Sciences Institute, Trinity College Dublin, Dublin 2, Ireland.,

^2^Dept. of Mechanical and Manufacturing Engineering, School of Engineering, Trinity College Dublin, Dublin 2, Ireland.

^3^Dept. of Mechanical, Aeronautical and Biomedical Engineering, University of Limerick, Ireland

^4^Advanced Materials and Bioengineering Research Centre, Trinity College Dublin & RCSI, Dublin 2, Ireland

*Correspondence: stavense@tcd.ie, [micorrig@tcd.ie](mailto:micorrig@tcd.ie), [gjohnson@tcd.ie](mailto:gjohnson@tcd.ie), [riffaulm@tcd.ie](mailto:riffaulm@tcd.ie), [dahoey@tcd.ie](mailto:dahoey@tcd.ie)

**Keywords**

Mesenchymal stem cell; Bone; Mechanobiology; Osteogenic Differentiation; Bioreactor

**
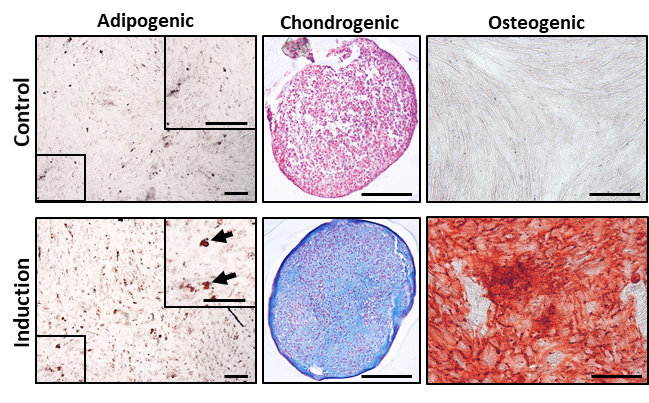
**

**Figure S1** Validation of trilineage potential of hBMSCs for adipogenesis (Oil red O, A), chondrogenesis (Alician Blue, B) and osteogenesis (Alizarin Red S, C) after 21 days in culture. Zoomed in images point to triglyceride accumulation in adipogenic conditions. Scale bar=200 µm


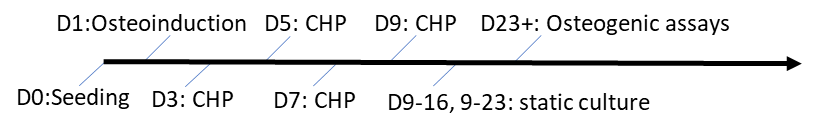


**Figure S2** Schematic of long term pressure mechanical stimulation regime.
